# Coping with heat: behavioural and physiological responses of savanna elephants in their natural habitat

**DOI:** 10.1093/conphys/cow044

**Published:** 2016-10-15

**Authors:** Michael A Mole, Shaun Rodrigues DÁraujo, Rudi J van Aarde, Duncan Mitchell, Andrea Fuller

**Affiliations:** 1 Conservation Ecology Research Unit, Department of Zoology and Entomology, University of Pretoria, Pretoria, South Africa; 2 Brain Function Research Group, School of Physiology, Faculty of Health Sciences, University of the Witwatersrand, Johannesburg, South Africa

**Keywords:** Behaviour, homeothermy, savanna elephant, thermal imaging, thermoregulation

## Abstract

Most of southern Africa's elephants inhabit environments where environmental temperatures exceed body temperature, but we do not know how elephants respond to such environments. We evaluated the relationships between apparent thermoregulatory behaviour and environmental, skin and core temperatures for tame savanna elephants (*Loxodonta africana*) that were free-ranging in the hot parts of the day, in their natural environment. Environmental temperature dictated elephant behaviour within a day, with potential consequences for fine-scale habitat selection, space use and foraging. At black globe temperatures of ~30°C, elephants adjusted their behaviour to reduce environmental heat load and increase heat dissipation (e.g. shade use, wetting behaviour). Resting, walking and feeding were also influenced by environmental temperature. By relying on behavioural and autonomic adjustments, the elephants maintained homeothermy, even at environmental temperatures exceeding 40°C. Elephants clearly have the capacity to deal with extreme heat, at least in environments with adequate resources of forage, water and shade. Future conservation actions should provide for the thermoregulatory, resource and spatial needs of elephants.

## Introduction

**Figure 1:**
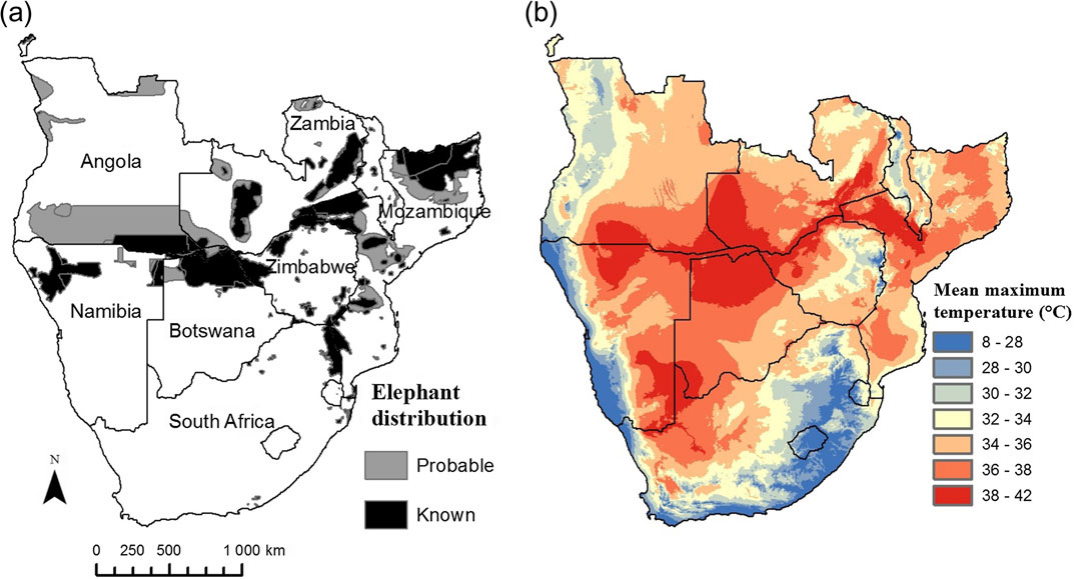
Maps of southern Africa illustrating: (**a**) known and probable present-day savanna elephant distribution (data source: www.elephantdatabase.org; accessed 2 June 2014 at 15.00 h); and (**b**) current mean maximal air temperatures (years 1950–2000; data source: www.worldclim.org; accessed 2 June 2014 at 14.00 h; Hijmans *et al*., 2005).

Iconic large mammals that flourish in Africa's hot and dry savannas cope well within the limits set by present thermal conditions, but may not do so when conditions become hotter and drier, as predicted with climate change (James and Washington, 2013). Predicting how they will respond to hotter and drier conditions, and implementing appropriate conservation measures if necessary, depends on us understanding how they are coping with the hottest environments currently. The majority of southern Africa's savanna elephants (*Loxodonta africana*) inhabit environments where maximal temperatures exceed their core body temperature (Fig. 1). Elephants lack sweat glands (Smith, 1980) and may have difficulty in thermoregulating when environmental temperatures exceed core body temperature (Williams, 1990; Phillips and Heath, 1995; Rowe *et al*., 2013). Studies on captive elephants have aided our understanding of elephant thermoregulation at moderate environmental temperatures (Kinahan *et al*., 2007a; Hidden, 2009; Weissenböck *et al*., 2010, 2012; Rowe *et al*., 2013), but we do not know how free-ranging elephants respond in extreme heat (~36°C). Whether elephants maintain homeothermy at environmental temperatures higher than core body temperature is also unknown.

Maintenance of thermal homeostasis requires elephants to balance the heat produced through metabolic activity and gained from the environment with heat loss to the environment. Elephants exposed in captivity to mild environmental temperatures can maintain thermal homeostasis through non-evaporative heat loss (Williams, 1990), but at higher environmental temperatures evaporative cooling is obligatory and takes place transcutaneously (Dunkin *et al*., 2013). It also has been proposed that elephants exposed to warm environments (below body core temperature) store heat, becoming heterothermic and conserving body water (Weissenböck *et al*., 2012). Most information on thermoregulation in savanna elephants, however, has been obtained from captive animals exposed to only moderate heat. To the best of our knowledge, there has been no previous study of thermoregulation in elephants in African savanna habitats, where air temperatures often exceed body temperature and where high solar radiation may add to the potential heat load on elephants.

In elephants, behaviours such as shade seeking (Kinahan *et al*., 2007b), changes in intensity of activity (Rowe *et al*., 2013) and water-related activities (e.g. mud bathing and swimming; Wright and Luck, 1984; Hidden, 2009; Dunkin *et al*., 2013) are likely to have thermoregulatory benefits, but may have various consequences (van Beest *et al*., 2012; van Beest and Milner, 2013; Long *et al*., 2014). For example, in large mammals, thermally induced behaviour may alter activity, habitat selection and fine-scale spatial use patterns (Aublet *et al*., 2009; van Beest *et al*., 2012; Owen-Smith and Goodall, 2014). As a result, feeding may decrease (Belovsky and Slade, 1986), and trade-offs in forage availability and quality may occur (van Beest *et al*., 2012; van Beest and Milner, 2013). Factors that alter behaviour and, consequently, spatial use patterns are ultimately important in dictating the impact elephants may have for other species (Guldemond and van Aarde, 2008) and for the survival of their own young (Young and van Aarde, 2010). Consequently, understanding how temperature drives elephant behaviour is important for the conservation and management of this species.

Here, we evaluate, for the first time, the associations between behaviours that appear to be thermally related and the environmental temperature, as well as the relationship between skin and core temperatures and environmental temperature, in tame savanna elephants that were free-ranging over the hottest part of the day in their natural environment. We also evaluate how skin and core temperatures are modified by an elephant's behaviour and assess the potential consequences of such behaviour.

## Materials and methods

### Study site and animals

The study took place close to Abu Camp (19°25′00.3″S; 22°35′03.6″E) in Botswana's Okavango Delta, from September to November 2012 (hot–dry season) and May to July 2013 (cool–flood season). The landscape consisted of seasonal swampland that included islands dominated by open savanna and bordered by narrow riverine forests. Floodwater was present throughout the study period.

This study was conducted on seven tame, partly free-ranging savanna elephants belonging to Abu Camp. The herd consisted of three adult females, three weaned calves (one male and two females) and one female suckling calf (for individual elephant characteristics, see Supplementary Data S1), which organized themselves into two family groups that spent most of the day separated. The elephants took part in tourism activities during the early morning and late afternoon. At night, they were housed in an open outdoor enclosure (50 m × 40 m), where they had access to food and water *ad libitum*. During the daytime, the elephants roamed unrestricted and were free to respond behaviourally to prevailing environmental conditions.

### Field protocol

During the daytime (between 09.00 and 16.00 h), two of us (M.A.M. and S.R.D.) selected a focal elephant randomly from the herd and followed it closely (10–50 m away) on foot as it roamed freely in its natural habitat. We recorded skin temperature (see next subsection) every 10 min, at times coinciding with environmental temperature measurements (see ‘*Environmental temperature*’ subsection below). At the same time, following a continuous focal sampling approach (Altmann, 1974), we recorded the behaviour of the focal elephant, by assigning its activity at the time to one of the following: walking, resting, feeding, drinking and wetting (swimming, mud bathing and spurting water over parts of body). We also recorded whether the elephant was exposed to the sun or in shade. The ethogram (Table 1) was similar to that of Guy (1976) and Leggett (2009). We considered a change in behaviour to have taken place when the subsequent behaviour lasted longer than 1 min. For a complete list of all the responses recorded, see Supplementary Data S2.

**Table 1:** Ethogram of behaviour recorded as part of the present study

Behaviour	Description
Walking	Moving at a constant rate from one point to another point
Resting	Standing or lying down while not engaged in any other behaviour. Includes sleeping
Feeding	Ingestion and/or handling of any plant material that leads to ingestion
Water-related activities	Any activity associated with water or mud, including dust bathing
Drinking	Ingestion of water
Wetting	Wetting the body by wallowing in mud or water, spraying mud or water over the body
Dust bathing	Throwing of dust over body
Other	Any unnatural behaviour or behaviour not associated with the above categories or relevant for this study, e.g. social interactions, fighting, playing, nursing
Shade utilization	More than 50% of body shaded

Walking, resting, feeding, any water-related activity and ‘other’ behaviour are mutually exclusive from one another. Shade utilization can occur simultaneously with any behaviour.

### Skin temperature

We recorded the skin temperature of the focal elephant, from an angle perpendicular to its sagittal plane, using a monopod-mounted infrared thermal camera (FLIR T640; FLIR Systems Inc., Portland, OR, USA) that was factory calibrated to record temperature with an accuracy of ±1.0°C. A calibration check was conducted before and after the study to make sure the camera was properly calibrated. This check involved taking thermal images of liquids at known temperatures. We supplied the camera with the following variables: emissivity = 0.98 (calculated for elephant skin); distance between camera and elephant = 10 m (unless specified otherwise); air temperature; relative humidity (measured using a portable psychrometer, Extech Instruments, ExTech^®^ HD500; Townsend West, Nashua, NH, USA) and reflected temperature. For supplementary information on how these parameters were measured, see Supplementary Data S3. For a given thermogram, we calculated the skin temperature for seven predefined body areas using hand-drawn complex polygons, in the software package FLIR Reporter Professional 9.0 (FLIR Systems Inc.).

### Core temperature

Elephant core temperature was recorded from the intestinal tract at 5 min intervals using miniature ingestible data loggers (iButton; Maxim Integrated Products, San Jose, CA, USA). Each data logger, when coated in inert, waterproof wax (Sasol, South Africa), measured 30 mm × 25 mm. We attached a brightly coloured satin ribbon, ~150 mm long, to the data logger to aid detection in dung. We ‘fed’ the data loggers to the elephants during early morning or evening by throwing the data logger into a stream of water that was flushed into the mouth from a hosepipe. Core temperature was not recorded in the two youngest elephants because they did not swallow the data loggers. Logger retention times in the gastrointestinal tract ranged between 19 and 145 h. Core temperatures were unstable while the data logger was still in the stomach (Hidden, 2009), so we analysed temperature data only after stabilization, defined by the first three identical consecutive measurements, 5 min apart. All data loggers were calibrated against a certified precision thermometer (Quat 100; Hereus, Hanau, Germany) in an insulated circulating water-bath, to a calibrated accuracy of 0.06°C.

### Environmental temperature

We recorded shaded air temperature and black mini-globe temperature (henceforth referred to as black globe temperature) at 5 min intervals using temperature-recording data loggers (iButton; Maxim Integrated Products; calibrated accuracy = 0.5°C). Mini-globe temperatures were recorded from the centre of a hollow copper sphere, 30 mm in diameter and painted matt black (Hetem *et al*., 2007). Globe temperature integrates the air temperature, radiation and wind speed to give an overall measure of the environmental thermal load on an animal. Mini-globe temperatures can be converted to standard (150 mm) globe temperatures if necessary (Hetem *et al*., 2007), but raw mini-globe temperatures served our purposes. The thermometers were mounted onto a portable custom-built weather station that was placed in an open area (exposed to solar radiation and wind), within 1 km of the focal elephant. The thermometers were placed 1.9 m above the ground, the average shoulder height of our elephants.

### Data analysis

We fitted generalized additive models (GAMs) with simple random effects (essentially mixed models) and generalized additive mixed models (GAMMs) to the data to model the association of each recorded behaviour, skin temperature and core temperature to various explanatory variables using the *mgcv* (Wood, 2006) and *nlme* (Pinheiro *et al*., 2013) packages in R (2.15.2; R Core team, 2012). A description of all the response and explanatory variables can be found in Supplementary Data S2. Air temperature and black globe temperature were correlated strongly (Spearman rank correlation, *r*_s_ = 0.96). We therefore retained only black globe temperature in our analyses because it incorporated convective and radiative aspects of the environment (Hetem *et al*., 2007).

The occurrence of each behaviour (walking, resting, feeding, drinking, wetting and shade use) at every 5 min interval was set as the response variable, and the associated black globe temperature, time of day, season, age class and family group were set as the explanatory variables. In a separate modelling step, the duration of time spent in shade per hour was set as the response variable, and the explanatory variables were mean black globe temperature, time of day, season, age class, family group and state. Recorded time intervals were rounded off to the nearest minute. Black globe temperatures were correlated weakly with time of day (*r*_s_ = 0.43, between 09.00 and 16.00 h). Time of day was therefore included as a potential explanatory variable for each response variable except skin temperature, as we had no *a priori* reason to expect that skin temperature would be dependent on time of day. Whether the elephant was wet or dry (‘state’) was added as an explanatory variable, because we expected that elephants that were wet or had recently used wetting behaviour might be less likely to seek shade. The behavioural responses of the suckling calf were not included in any of the models; the calf spent most of the day accompanying its mother.

To model the response of skin temperature, the skin temperature recorded at 10 min intervals served as the response variable, and associated black globe temperature, age class, and state (exposed to sun, in shade, wet) served as potential explanatory variables. To model the response of core temperature, the core temperature recorded at 5 min intervals served as the response variable, and associated black globe temperature, time of day, age class and state served as potential explanatory variables.

We used GAMMs to model the response of hourly durations of shade use, skin temperature and core temperature, and GAMs with simple random effects to model the behavioural binary responses (Wood, 2006). To account for repeated observations on individuals, in all models, individual elephant identity was entered as a random effect structure (Wood, 2006) or as a simple random effect by treating the random effect as a smooth term (Wood, 2008). For skin temperature models, we included part of the body as a nested random effect within each elephant to account for differences in skin temperature on different parts of the body. We formulated each set of candidate models using an all-subset approach that comprised all possible combinations of the relevant explanatory variables without interactions. Interactions were excluded from the analytical approach because of our low sample size of elephants (the inclusion of interaction terms in some cases would have resulted in smoothed responses being modelled based on data obtained from one individual of the herd). The area under the receiver operating characteristic (ROC) curve was calculated to assess the accuracy and performance of each binary response GAM (Fielding and Bell, 1997). The ROC values vary between 0.5 (discriminating power not better than chance) and 1 (perfect discriminating power). Models with ROC values ≥0.7 were considered to have acceptable discriminating power (Hosmer and Lemeshow, 2004). Adjusted $R^{2}$ values were calculated to assess the measure of fit for each GAMM. For model selection, we ranked each candidate mixed model using Akaike's information criterion (AIC; Burnham and Anderson, 2002). The strength of support for the best model and alternativee best models was assessed using AIC differences $\left({∆\mathrm{AIC}}_{i}\right)$ between the approximate best model $\left({∆\mathrm{AIC}}_{i}\;=\;0\right)$ and alternativee candidate models. The Akaike weight $(w_{i})$ for each candidate model was also calculated (Burnham and Anderson, 2002), and from this, we were able to assess further the relative importance of each explanatory variable by summing the Akaike weights $(w_{i})$ across all candidate models in which the particular variable appeared. This was achievable because the variables appeared an equal number of times within each subset of candidate models. These values ranged between 0 and 1; the larger the value the more important the variable was relative to other variables within the set of candidate models (Burnham and Anderson, 2002). Given that candidate models with ${∆\mathrm{AIC}}_{i}$ < 2 are considered as good as the best model and have substantial support as an alternative best model (Burnham and Anderson, 2002), the analysis of relative importance strengthened the interpretation of our results, particularly in the event of model uncertainty or when there was no substantial support for a best model based on AIC differences.

To view the relationship between response and explanatory variables, we plotted the partial response curves showing the relationship of the partial residuals of the response variable on the linear predictor scale and the relevant explanatory variables of the best approximate model. Plots were centred to have a mean value of zero along the *y*-axis, and the trends rather than the actual values of the plots were used to describe the responses to the smoothed explanatory variables.

## Results

### Environmental temperatures

During both seasons, black globe temperature followed a consistent 24 h pattern, increasing after sunrise, peaking at ~14.00 h and decreasing thereafter. Black globe temperature ranged between 11 and 50°C during the hot–dry season (74 days) and between 5 and 42°C during the cool–flood season (55 days). A standard *t*-test revealed that mean maximal black globe temperature was significantly higher ($t_{127}$ = 8.4, *P* < 0.0001) during the hot–dry season (mean ± SD = 42.6 ± 4.6°C) than during the cool–flood season (34.7 ± 6.2°C). Likewise, mean minimal black globe temperature was significantly lower ($t_{127}$ = 14.6, *P* < 0.0001) during the cool–flood season (11.4 ± 2.1°C) than during the hot–dry season (18.1 ± 2.9°C). Importantly, 50% of the black globe temperature recordings between the observational hours of 09.00 and 16.00 h were above 36°C.

### Behavioural responses

We recorded behaviour for 545 h over 80 days (overall sampling effort in days: adult female 1 = 14, adult female 2 = 19, adult female 3 = 14, weaned female 1 = 11, weaned female 2 = 11 and weaned male 1 = 11). Overall, the elephants spent most of their time between 09.00 and 16.00 h feeding (mean ± SD = 85.0 ± 6.8%), and very little time engaged in walking (6.5 ± 2.8%), resting (1.7 ± 2.7%), wetting (3.0 ± 3.5%) and drinking (1.5 ± 0.7%). Dust bathing occurred infrequently and, therefore, was not included in the analyses. The elephants were in shade for 29.5 ± 18.1% of the time.

Owing to high model uncertainty within each modelled behavioural response (low Akaike weights for approximate best model; Table 2; see Supplementary Data S4 Tables A–G for a full list of candidate models), model interpretation relied mostly on the estimated parameter weights for each set of candidate models (Table 3). Overall, model accuracy and predictability of full models was acceptable for the probability of resting (ROC = 0.79) and wetting (ROC = 0.76). The full model for the probability of shade use was also acceptable (ROC = 0.74), and a strong fit was observed in the full model for the duration of shade use (adjusted *R*^2^ = 0.53). Model accuracy and predictability were relatively poor for the probability of drinking (ROC = 0.65), walking (ROC = 0.62) and feeding (ROC = 0.64).

**Table 2:** Summary of selected best (∆AIC_i_ = 0) generalized additive mixed models from each set of candidate models

Response	Best candidate model_i_	*K*	*w*_*i*_	ROC	*R*^2^
Probability of walking	Black globe temperature* + time* + group*	9.5	0.52	0.62	n/a
Probability of resting	Black globe temperature*	7.5	0.10	0.79	n/a
Probability of drinking	Black globe temperature + time*	4.3	0.21	0.64	n/a
Probability of wetting	Black globe temperature* + time* + season*	15.4	0.26	0.76	n/a
Probability of shade use	Black globe temperature* + group*	9.5	0.10	0.74	n/a
Probability of feeding	Black globe temperature* + time + season*	12.2	0.47	0.64	n/a
Duration of shade use	Black globe temperature* + state* + group*	7	0.37	n/a	0.54
Skin temperature	Black globe temperature* + state* + age class	10	0.86	n/a	0.36
Core temperature	Black globe temperature + time* + age class	8	0.49	n/a	0.31

*Model parameter coefficient significant (*P* < 0.05).

**Table 3:** Summary of the relative importance of each explanatory variable in each set of candidate generalized additive mixed models and the area under the receiver operating characteristic curve of each full model, for each response modelled

Variable	Probability walking	Probability resting	Probability drinking	Probability wetting	Probability feeding	Probability shade use	Duration shade use
Black globe	**1.0**	**1.0**	0.67	**1.0**	**1.0**	**1.0**	**1.0**
Time of day	**0.99**	0.44	**1.0**	**1.0**	0.92	0.35	0.15
Season	0.31	0.33	0.38	**0.98**	**0.97**	0.48	0.30
Family group	**0.77**	0.47	n/a	0.49	n/a	**0.54**	**0.94**
Age class	n/a	0.48	0.48	0.48	0.47	0.48	0.33
State	n/a	n/a	n/a	n/a	n/a	n/a	**1.0**
ROC	0.62	0.79	0.65	0.76	064	0.74	0.53*

The relative importance of each explanatory variable was assessed and ranked by summing the Akaike weights $(w_{i})$ across all candidate models in which the particular variable appeared. Between 0 and 1, the larger the value the more important the variable is relative to other variables within the set of candidate models (Burnham and Anderson, 2002). Variables significant in at least one of the selected best or alternative best models are shown in bold. Variables not included in the analysis for a particular behaviour are illustrated as n/a. ROC, receiver operating characteristic. *Adjusted *R*^*2*^ value, not ROC value.

**Figure 2:**
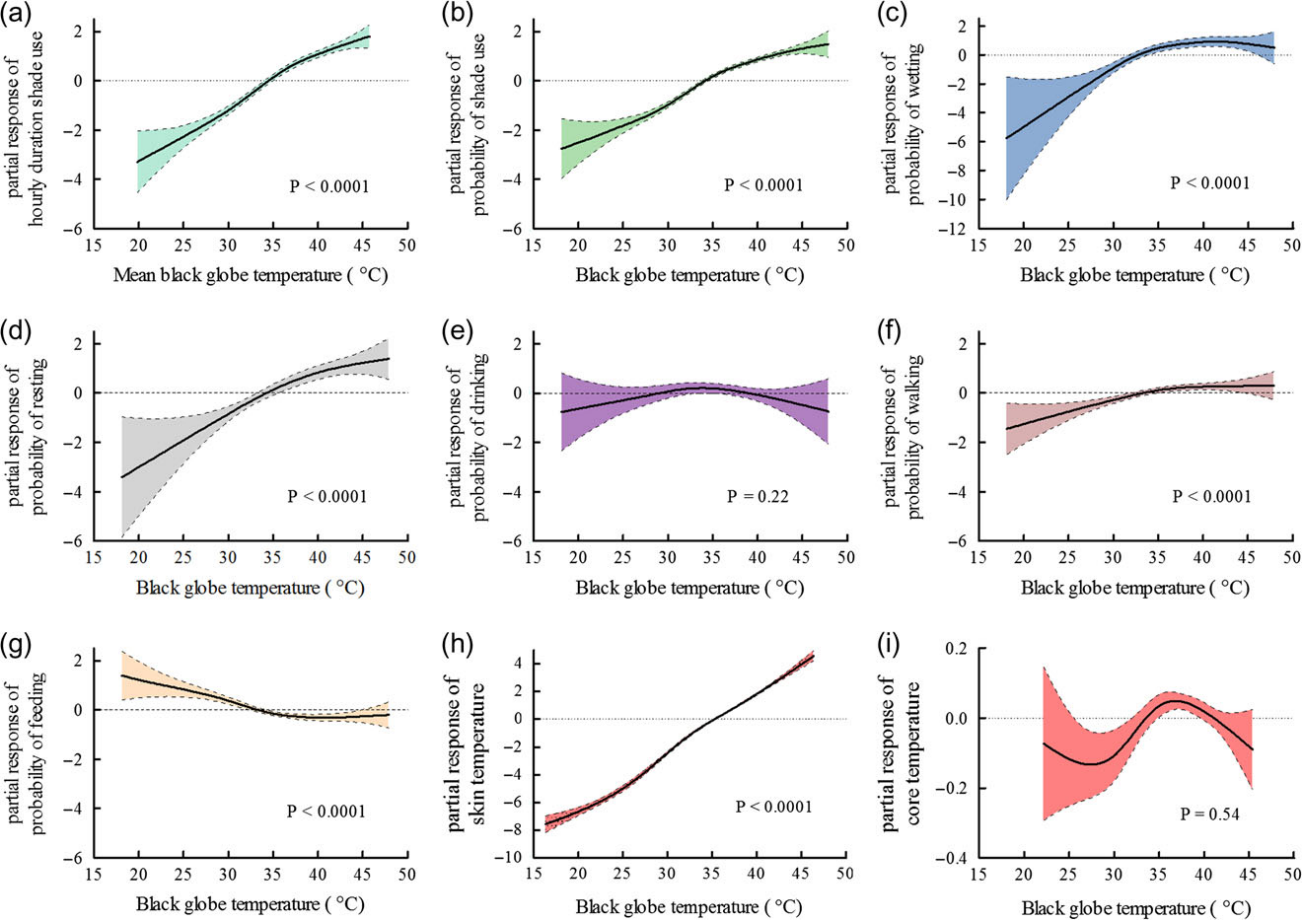
Outcomes of generalized additive mixed model analysis for recorded behaviours (**a–g**), skin (**h**) and core temperatures (**i**) dependent on black globe temperature in selected best approximate models. The *y*-axes are partial residual responses plotted on the scale of the linear predictor; they represent how the response would deviate from the predictions of a model that assumed the response was independent of the *x*-axis variable, here black globe temperature. Shaded areas and dashed lines represent 95% confidence intervals.

**Figure 3:**
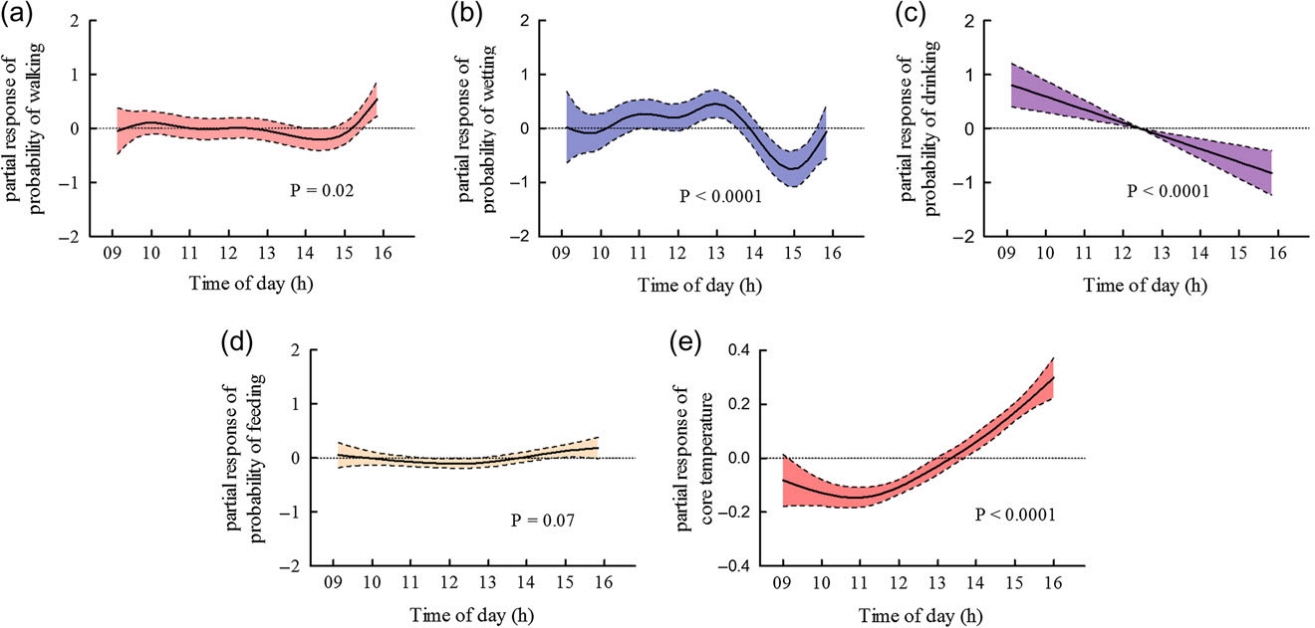
Outcomes of generalized additive mixed model analysis for recorded behaviours (**a–d**) and core temperature (**e**) dependent on time of day in selected best approximate models. Shaded areas and dashed lines represent 95% confidence intervals.

The probability of elephants being in shade and the amount of time that they spent in shade were explained best by black globe temperature and to some degree by family group (Tables 2 and 3). The likelihood and duration of being in shade increased significantly with black globe temperature, and elephants were most likely to be in shade at black globe temperatures >35°C (Fig. 2a and b). The likelihood of being in shade (*P* < 0.0001) as well as how long elephants spent in shade (*P* = 0.017) were both greater in the family group with the suckling calf than in the family group without the calf. How long the elephants were in shade was also dependent on whether they were wet or dry (state; Table 3), with dry elephants being in shade for longer (*P* < 0.0001). Just as the elephants were most likely to be in the shade at black globe temperatures >35°C, so too were they most likely to rest (Fig. 2d). Black globe temperature was the only variable that explained the probability of resting (Table 3), and resting increased significantly with black globe temperature. Family group had no significant effect on the probability of resting but had a strong influence on walking behaviour (Table 2), with the family group hosting the calf having a lower likelihood of walking than did the family group without the calf (*P* < 0.0001). The probability of walking was also influenced strongly by black globe temperature and time of day (Table 3). Walking increased significantly with black globe temperature (Fig. 2f) and was more likely to occur later in the day (between 15.00 and 16.00 h; Fig. 3a).

The probability of wetting (swimming, mud bathing and spurting water over parts of body) was explained best by black globe temperature, time of day and season (Table 3). Wetting increased significantly with black globe temperature and was most likely to take place when black globe temperature exceeded 33°C (Fig. 2c). The elephants were more likely to undertake wetting behaviour during the hot–dry season than during the cool–flood season (*P* = 0.003). There also was an increased likelihood of wetting taking place around 13.00 h in the day (Fig. 3b).

Time of day was by far the strongest influence on when the elephants drank (Table 3). The probability of drinking peaked during the morning hours and then decreased throughout the day (Fig. 3c). Black globe temperature was also included in the approximate best model (Table 2); however, the parameter weight and the partial response curve for black globe temperature suggest that it did not have a strong influence on drinking behaviour (Table 3 and Fig. 2e).

Time of day was not the strongest predictor of feeding (Table 3). Although time of day contributed as an explanatory variable with a high parameter weight (Table 3), feeding was only slightly less likely to occur around midday (Fig. 3d; *P* = 0.07). The probability of feeding was explained better by black globe temperature and by season (Table 3). Feeding decreased significantly with black globe temperature and was more likely to take place at temperatures <33°C than >33°C (Fig. 2g). The likelihood of feeding also was less during the hot–dry than during the cool–flood season (*P* = 0.003).

### Skin temperature

We recorded skin temperature over 36 days during the hot–dry season and over 28 days during the cool–flood season (overall sampling effort in days: adult female 1 = 11, adult female 2 = 8, adult female 3 = 11, weaned female 1 = 6, weaned female 2 = 9, weaned male 1 = 8 and suckling calf 1 = 12). Between 09.00 and 16.00 h, skin temperature ranged between 20.2 (mean minimum = 27.5 ± 3.6°C) and 42.4°C (mean maximum = 40.7 ± 2.9°C) for all elephants (*n* = 7) during the hot–dry season (mean = 34.1 ± 3.0°C) and between 18.5 (mean minimum = 20.1 ± 4.4°C) and 40.3°C (mean maximum = 39.1 ± 1.4°C) during the cool–flood season. Of seven candidate GAMMs (see Supplementary Data S4 Table H for full list of candidate models), a single GAMM described the variation in skin temperature best, with 86% model certainty (*w*_*i*_ = 0.86; Table 2). Black globe temperature, state and age class were included as explanatory variables in this model, which described up to 36% (adjusted *R*^2^ = 0.36) of the variation in skin temperature. Skin temperature increased significantly as a function of black globe temperature (*P* < 0.0001; Fig. 2h) and was significantly lower when elephants were in shade (*P* < 0.0001) or bathed (*P* < 0.0001) than when they were exposed to direct sunlight. Age class was added as an explanatory variable in the model, but skin temperatures of suckling (*P* = 0.10) and weaned calves (*P* = 0.14) were not significantly different from those of adults.

### Core temperature

We recorded 18 days of core temperature for five elephants during the hot–dry season (adult female 1 = 6 days, adult female 2 = 4 days, adult female 3 = 2 days, weaned female 1 = 1 day and weaned male 1 = 5 days) and 8 days of core temperature for two elephants during the cool–flood season (adult female 1 = 3 days and adult female 3 = 5 days). Core temperature followed a consistent 24 h pattern, increasing during the daytime from ~08.00 until ~18.00 h, followed by a decrease during the night. This pattern was consistent in all elephants. Core temperature ranged between a mean minimum of 36.0 ± 0.3°C and a mean maximum of 37.1 ± 0.6°C during the hot–dry season, and between a mean minimum of 35.9 ± 0.5°C and mean maximum of 37.0 ± 0.3°C during the cool–flood season. The 24 h mean core temperature range (the difference between the maximal and minimal core temperature over a 24 h period) for all elephants was 1.2 ± 0.4°C during the hot–dry season and 1.1 ± 0.5°C during the cool–flood season.

Of 15 candidate GAMMs (see Supplementary Data S4 Table I for full list of candidate models), the most plausible model described the variation in core temperature with 49% model certainty (*w*_*i*_ = 0.49; Table 2). Black globe temperature, time of day and age class were included as explanatory variables and described up to 31% (adjusted *R*^2^ = 0.31) of the variation in core temperature. However, the partial response curve implies that black globe temperature did not influence core temperature (*P* = 0.54; Fig. 2i), nor was there a significant difference between age classes (*P* = 0.08). Core temperature, however, did increase significantly (*P* < 0.0001; Fig. 3e) as a function of time of day between 09.00 and 16.00 h, the time over which we recorded behaviour and skin temperature.

## Discussion

Our study is the first to relate skin and core body temperatures in elephants with environmental temperature and to establish how behaviour might assist free-ranging elephants to thermoregulate. Our results inform our understanding of elephant thermoregulation and enhance our understanding of the interaction between elephants and their natural environment.

### Environmental heat load dictated elephant behaviour

Our elephants altered their behaviour by seeking shade, increasing wetting behaviour or resting more, either in the shade or in the sun, when environmental heat load was high. High environmental heat load also was associated with less feeding and more walking. We concede that the association between walking and heat load may have arisen, at least in part, from instinctive movement towards meeting points for the afternoon tourist activities, which began at ~17.00 h; afternoon heat load tended to be high. Nevertheless, we suggest that, in hot environments, environmental heat load will be a strong determinant of habitat selection and temporal patterns of space use by elephants. However, not all thermally related behaviour was driven by environmental heat load. Specific behavioural activities were also dependent on time of day. Drinking, in particular, was more likely to occur in the morning hours than at other times of day, independent of environmental heat load. We suspect that an endogenous behavioural rhythm determined when our elephants chose to drink. Also, some behaviours cannot take place simultaneously, and there may be some interaction between behaviours as a result. For example, increased walking may have resulted in a decrease in wetting in the late afternoon.

### Elephants maintained homeothermy under positive heat load

Amongst the factors that we measured, the strongest determinant of core body temperature was time of day (Fig. 3e). Neither we nor anyone else has yet measured the nychthemeral rhythm of core body temperature in free-living elephants, but we believe that the dependence of core body temperature on time of day was part of that rhythm. In spite of their huge thermal inertia, elephant core temperatures were not constant, but rather exhibited strong 24 h rhythms when elephants were confined in open outdoor enclosures at night (our elephants and Hidden, 2009) or when confined to enclosures over 24 h (Kinahan *et al*., 2007a). The trends that we observed between 09.00 and 16.00 h were consistent with reported 24 h rhythms. With the pattern so regular, we could average the core temperature over our observation period and explore what influenced that average. Black globe temperature did not drive this average, even though black globe temperature reached ≥36°C for 40% of the recordings, so that elephants in exposed locations would have been under positive heat load. We have shown, therefore, for the first time, that savanna elephants can maintain homeothermy, with body temperature fluctuating by no more than 1.5°C over 24 h, under positive radiant and convective heat load.

### Thermally related behaviour benefitted thermoregulation

Excluding behavioural adjustments, evaporative cooling is the only means for elephants to dissipate heat to the environment when environmental temperatures exceed body temperature, as occurred frequently in the hot–dry season. Indeed, evaporative cooling is obligatory in elephants at much lower environmental temperatures too (Dunkin *et al*., 2013). Wetting of the skin, which occurred most often in the hot–dry season, enhances cutaneous evaporative water loss (Dunkin *et al*., 2013), but took place only sporadically. When their skins were not wetted externally, the elephants would have had to rely on water transported through the skin for evaporative cooling (Wright and Luck, 1984; Dunkin *et al*., 2013), necessarily depleting body water stores and disrupting osmoregulation. Our elephants always had access to drinking water to redress that depletion, but not all savanna elephants do (de Beer *et al*., 2006). It is highly likely that savanna elephants, as documented in other large mammals (Fuller *et al*., 2014), have evolved mechanisms for reducing the demand for evaporative cooling when exposed to high environmental temperatures, and our study is the first to show that indeed they have done so. Our elephants exploited the shade available in their habitat to reduce their ambient heat load, and both the probability of being in shade and the duration of periods in shade were controlled behavioural responses correlated with the heat load to which they would have been exposed if they had not sought shade (Figs 2a and b and Table 3). Also controlled was the propensity to rest as environmental heat load increased (Fig. 2d and Table 3), especially at the highest loads. Resting avoided the need to dissipate the extra metabolic heat that the elephants would have generated if they had exercised to the extent that they did in cooler environments (Rowe *et al*., 2013). That suite of behavioural responses, together with evaporative cooling, allowed them to attain the homeothermy that we have demonstrated, in the hottest environments in which elephants have been studied.

Being in the shade and wetting of the skin reduced the skin temperatures of our elephants below those experienced when they were in the sun. Skin temperature is determined primarily by ambient conditions, wetness of the skin and vasomotor state. Having a low skin temperature is not beneficial for ameliorating the thermal status of animals in hot environments. In environments sufficiently cool such that the animal can dissipate heat by convection and radiation, a low skin temperature reduces the temperature gradient driving heat loss. In environments sufficiently hot for radiation and convection to impose a heat load on the animal, a low skin temperature increases the temperature gradient driving the heat gain. Mammals therefore vasodilate in hot environments; vasodilatation increases the rate of heat transfer from the body core to the periphery and also elevates skin temperature. We believe that our elephants vasodilated progressively as their environment got hotter (Williams, 1990), even when they were in the shade and when their skin was wet externally. Their skin temperature increased as a function of the heat load in exposed parts of their habitat (Fig. 2h). Given that the core temperature did not increase with heat load at very high environmental heat loads (Fig. 2i), it must have been progressive activation of temperature receptors in the skin that elicited the behavioural responses.

If elephants are to seek shade and wet their skins at high environmental temperatures, they need access to shady trees and to water; they also need water to replace that lost in evaporative cooling. On hot days (>35°C), our elephants spent up to 60% of the day in shade. Water-related activity occupied up to 10% of the day's activities. Our elephants did not seek shade more often nor did they spend longer in the shade in the hot–dry season than they did in the cool–flood season, but there was a higher probability of wetting during the hot–dry season than during the cool–flood season. Elephants may be constrained to forage close to water when environmental temperatures are high. If elephants forage close to water when environmental temperatures are high, and can therefore readily replace water lost from body fluids and can wet their skins, they might not need to resort as much to seeking shade and to resting to maintain homeothermy.

### Homeothermy is costly but is a high-priority option for elephants at high environmental temperatures

For how long elephants must feed to meet their nutritional needs is unknown; it is likely to depend on the availability and nutritional quality of forage. Our elephants spent 60–90% of the observation period feeding; behaviour consistent with that recorded in previous studies, where elephants spent up to 12–16 h a day feeding (Wyatt and Eltringham, 1974; Guy, 1976). However, feeding decreased with an increase in environmental temperature, especially at black globe temperatures above core temperature (Fig. 2g). The elephants then increased resting and sought shade or opportunities to wet their skin through mud bathing and swimming. Although our elephants sometimes did eat while in the shade, in general those behaviours, all related to maintenance of homeothermy, would compete with feeding and, potentially, prevent the elephants meeting their nutritional needs. Elephants must trade off the benefits of high-quality resources against the costs of accessing them (Harris *et al*., 2008). Either because foraging in the sun imposed too great a demand on body water reserves or because the energetic costs of being active in direct sunlight increased unacceptably (Rowe *et al*., 2013), the elephants at high environmental temperatures assigned maintenance of homeothermy a higher priority than they did feeding.

To compensate for feeding time lost, elephants may increase foraging efficiency (rate of food intake) or feed at cooler times of the day or night (as do other large herbivores: Maloney *et al*., 2005; Hetem *et al*., 2012). However, our elephants were not free to forage at night, and their need to maintain nutrition might explain why they spent less of the daytime resting than did other elephants (Guy, 1976; Leggett, 2009). Furthermore, our elephants were not limited in terms of resources. Recent studies investigating displacement activities in elephants imply that elephants indeed are more active at night than during the day when environmental temperatures are high (Loarie *et al*., 2009; Leggett, 2010), suggesting that wild elephants are also adjusting behaviour and spatial use patterns in response to environmental temperatures. Whether trade-offs between maintaining different homeostatic systems arise in more extreme environments where resources such as food and water are limited warrants further investigation.

### Conclusion

From our study, it is clear that climate dictates elephant behaviour and, therefore, may have consequences for their ecology. Environmental temperature is a significant factor dictating elephant behaviour and is likely to be a key determinant of habitat selection and space use in elephants. We therefore cannot ignore the consequences of climate when dealing with the conservation of elephants.

Elephants clearly have a broad behavioural capacity to deal with extreme heat and, if given access to adequate resources of forage, water and shade, can maintain homeothermy. However, whether or not elephants can cope with thermal stress in a resource-limited environment remains to be seen. We know that the availability of food and surface water has consequences for elephant distribution, reproduction and, ultimately, survival (Foley *et al*., 2008; Trimble *et al*., 2009; Young and van Aarde, 2010). Yet, the interacting effects of thermal stress in such environments need to be addressed in future studies. How much elephants are able to rely on behavioural adjustments before there are detrimental physiological effects or whether trade-offs between nutritional needs and thermal needs come into play is unknown. It is plausible that an elephant's ability to buffer extreme climatic conditions behaviourally without costly trade-offs is likely to be hindered if resources such as shade, water and forage become limited. However, if elephants are given the space and the opportunity to seek out more suitable habitats during thermally stressful periods, then these potential trade-offs may be alleviated.

## Supplementary Material

Supplementary Data
