# Supplementary material for: Coping with heat: behavioural and physiological responses of savanna elephants in their natural habitat
Source: Conserv Physiol. 2016 Oct 15;4(1):cow044. doi: 10.1093/conphys/cow044 (PMC5066386; doi:10.1093/conphys/cow044)
Supplement: Supplementary Data [file cow044_conphys-2016-013.pdf]

Supplementary Data S1-S4

S1: Identity, sex, age, family group association and body measures of savanna elephants in the present study.

S2: Response and explanatory variables used in the analytical modelling.

S3: Summary of methods used to measure the parameters needed to record skin temperature on the elephants in the present study.

S4: Summaries of candidate generalised additive mixed models for all behavioural responses and responses of skin and core temperature to a number of explanatory variables (variables listed in S2).

S1. Identity, sex, age, family group association and body measures of savanna elephants in the present study.

ID	Elephant name	Sex	Age	Age class	Mass (kg)	Shoulder height (m)	Family Group
Adult female 1	Cathy	Female	47	Adult	3510	2.5	A
Adult female 2*	Shireni	Female	28	Adult	2690	2.4	A
Adult female 3♦	Kitty	Female	18	Adult	2140	2.36	B
Weaned male 1*	Abu	Male	7	Weaned calf	1740	2.18	A
Weaned female 1♦	Lerato	Female	4	Weaned calf	1100	1.86	B
Weaned female 2	Paseka	Female	3	Weaned calf	900	1.64	A
Suckling calf 1*	Warona	Female	1	Suckling calf	320	1.34	A

*Weaned male 1 and suckling calf 1 are the offspring of adult female 2; ♦Weaned female 1 is the offspring of adult female 3.

S2. Response and explanatory variables used in the analytical modelling.

Variables	Type	Description
**Response variables**		
Probability of walking	Binary	**1**: walking taking place
		**0**: no walking taking place
Probability of resting	Binary	**1**: resting taking place
		**0**: no resting taking place
Probability of foraging	Binary	**1**: foraging taking place
		**0**: no foraging taking place
Probability of drinking	Binary	**1**: ingestion of water taking place
		**0**: no ingestion of water taking place
Probability of wetting	Binary	**1**: mud bathing/splashing/swimming taking place
		**0**: no wetting of any sort taking place
Probability of shade use	Binary	**1**: >50% of the body exposed to shade
		**0**: >50% of the body exposed to direct sun
Duration of shade use	Proportional	**0-100%**: percentage of time spent in the shade per hour
Skin temperature	Continuous	Surface temperature recordings at 10 minute intervals (°C)
Core temperature	Continuous	Intestinal body temperature recordings at 5 minute intervals (°C)
**Explanatory variables***		
Black globe temperature	continuous	Black mini-globe temperature (ºC), as an index of environmental temperature, measured at 5-minute intervals.
Time of day (*time*)	continuous	Each fifth minute interval (Binary behaviour models and core)
		Hour of day (Duration of shade use models)
Family group (*group*)	categorical	**A**: 5 individuals; 2 adults, 2 weaned calves, 1 suckling calf
		**B**: 2 individuals; 1 adult and 1 weaned calf
Age class (*class*)	categorical	**Adult**: Individuals over 11 years old
		**Weaned calf**: Individuals 3-7 years old
		**Suckling calf**: Individual 3 years and younger
Season	categorical	**Hot-dry season**: Observations during September to November
		**Cool-flood season**: Observations during May to July
State	categorical	**Dry**: elephant remained dry for entire hour of observation
		**Wet**: Wetting took place within the first 45min of the hour

Notes: * names in italics are acronyms or abbreviations used throughout analysis

S3: Summary of methods used to measure the parameters needed to record skin temperature on the elephants in the present study.

We recorded skin temperature using an infrared thermal camera (FLIR T640, FLIR Systems Inc., Portland, Oregon, USA), which was mounted on a monopod. The camera had a 25˚ lens, with a 7.5–14 μm spectral range and was factory (FLIR Systems Inc., Portland, Oregon, USA) calibrated to record temperature with an accuracy of ± 1.0˚C. The camera detects infrared radiation emitted from the surface of an object, which is converted into a temperature value. These values are depicted in the form of an infrared thermal image (thermograph). Each thermograph is 640 × 480 (307 200) pixels where each pixel represents a temperature value.

The radiation that is detected by the camera originates from three different sources; radiation from the target object, reflected radiation from the object's surroundings and radiation from the surrounding air. Therefore, the camera output can be described as follows:

$$W_{tot} = \varepsilon.\tau.W_{obj} + (1-\varepsilon).\tau.W_{amb} + (1-\tau).W_{atm}$$

where ε is the emissivity of the target object, τ is the transmittance of the atmosphere, W_{obj} is the amount of radiation emitted from the target object, W_{amb} is the amount of radiation emitted from the object's surroundings and W_{atm} is the amount of radiation present in the surrounding air. To ensure accurate skin temperature recordings we supplied the camera with the following parameters: emissivity=0.98, distance=10 m (unless specified otherwise), air temperature, relative humidity and reflected temperature.

Emmisivity

The amount of radiation emitted from an object comes from two major sources; radiation from the object itself (emissivity, ε) and radiation from the surrounding environment that is reflected off of the object (reflectivity, ρ). This is described as follows:

$$\varepsilon + \rho = 1$$

To calculate the emissivity of elephant skin, we placed an object of known emissivity (black scotch tape, ε=0.95) onto a piece of elephant skin. Both the skin and tape were cooled to the same temperature (5˚C). The emissivity of the camera was adjusted to 0.95 and the surface temperature of the black tape was recorded. We then adjusted the emissivity of the camera until the temperature of the elephant skin was identical to the temperature of the tape. The emissivity at which the skin temperature and the tape temperature were identical represented the emissivity of elephant skin. This value was 0.98, which is similar to the emissivity of human skin. Therefore, 98% of the radiation emitted from elephant skin is radiation from the skin itself and 2% is from reflected radiation. An emissivity of 0.98 was used for elephant skin throughout the study.

Distance

The amount of radiation emitted from the air surrounding the target object is dependent on the volume of air between the object and the observer (Wolfe and Zissis 1989). Therefore, we supplied the camera with the distance between the camera and the focal elephant. We consistently maintained a distance of 10 m to avoid sampling error. Where it was not possible to maintain this distance, we visually estimated the distance and supplied this to the camera.

Air temperature and relative humidity

The amount of radiation emitted from the air surrounding the target object is also dependent on the temperature and water content of the air (Wolfe and Zissis 1989). Therefore, we

supplied the camera with ambient temperature and relative humidity that we recorded using a portable psychrometer (ExTech® HD500, Townsend West, Nashua, U.S.A).

Reflected temperature

To account for reflected radiation from the environment, we calculated the reflected temperature by setting emissivity to 1.00 and distance to 0 m, before obtaining a thermal image of a diffuse reflector. The diffuse reflector comprised of a wrinkled sheet of aluminium foil placed over a spherical polystyrene ball (250 mm in diameter). Aluminium foil has an emissivity of 0.04. Therefore 99.6% of radiation from the surrounding environment is reflected from the aluminium foil. By setting ε to 1.00, we assumed that all radiation from the surrounding environment was represented by the average temperature of the diffuse reflector. We then substituted this average temperature into the camera parameters before obtaining a thermograph of the focal elephant. This ensured that skin temperature measurements excluded all reflected radiation from the surrounding environment.

Reference:

Wolfe WL, Zissis GJ (1989) *The infrared handbook*. Department of the Navy, Washington, D.C.

S4 Table A. Summary of candidate generalised additive mixed models for the probability of wetting.

no.	$model_i$	K	LogLik	AIC_i	ΔAIC_i	w_i	ROC
1	Tmg + time + season	15.4	-1066.0	2162.91	0	0.26	0.757
2	Tmg + time + group + season	15.6	-1065.9	2162.98	0.07	0.25	0.757
3	Tmg + time + class + season	15.7	-1065.9	2163.06	0.15	0.24	0.757
4	Tmg + time + group + class + season	15.8	-1065.8	2163.17	0.25	0.23	0.757
5	Tmg + time	14.3	-1071.0	2170.63	7.71	0.01	0.751
6	Tmg + time + group	14.5	-1070.9	2170.73	7.82	0.01	0.751
7	Tmg + time + class	14.7	-1070.7	2170.83	7.91	0.00	0.752
8	Tmg + time + group + class	14.8	-1070.7	2170.96	8.05	0.00	0.752
9	Tmg + + season	9.7	-1085.6	2190.61	27.7	0.00	0.730
10	Tmg + group + season	9.8	-1085.5	2190.69	27.78	0.00	0.730
11	Tmg + class + season	9.9	-1085.5	2190.76	27.84	0.00	0.730
12	Tmg + group + class + season	10.1	-1085.4	2190.87	27.96	0.00	0.730
13	Tmg	8.6	-1092.6	2202.33	39.42	0.00	0.720
14	Tmg + group	8.7	-1092.5	2202.44	39.53	0.00	0.720
15	Tmg + class	8.9	-1092.4	2202.52	39.61	0.00	0.720
16	Tmg + group + class	9.0	-1092.3	2202.66	39.75	0.00	0.720
17	time + season	12.2	-1091.3	2207.06	44.15	0.00	0.736
18	time + group + season	12.4	-1091.2	2207.13	44.22	0.00	0.737
19	time + class + season	12.4	-1091.2	2207.17	44.26	0.00	0.736
20	time + group + class + season	12.5	-1091.1	2207.27	44.36	0.00	0.737
21	season	6.4	-1117.7	2248.36	85.44	0.00	0.699
22	group + season	6.6	-1117.7	2248.43	85.52	0.00	0.700
23	class + season	6.6	-1117.6	2248.47	85.56	0.00	0.700
24	group + class + season	6.7	-1117.6	2248.57	85.66	0.00	0.700
25	time	11.2	-1146.6	2315.48	152.56	0.00	0.662
26	time + group	11.3	-1146.5	2315.57	152.65	0.00	0.663
27	time + class	11.4	-1146.4	2315.64	152.73	0.00	0.662
28	time + group + class	11.6	-1146.3	2315.76	152.84	0.00	0.662
29	group	5.4	-1172.4	2355.57	192.66	0.00	0.600
30	class	5.5	-1172.3	2355.65	192.74	0.00	0.600
31	group + class	5.7	-1172.2	2355.77	192.86	0.00	0.600

Notes: For each model, the model number (no.), number of parameters (K), loglikelihood value (LogLik), Akaike information criteria (AIC_i), difference in AIC between the best fit model and $model_i$ (ΔAIC_i), Akaike weight (w_i), area under receivers operating characteristic curve (ROC) are shown. Parameters: Tmg = black mini-globe temperature; time = time of day; class= age class; group = family group.

S4 Table B. Summary of candidate generalised additive mixed models for the probability of shade use.

no.	$model_i$	K	LogLik	AIC_i	ΔAIC_i	w_i	ROC
1	Tmg + group	9.5	-3459.0	6936.85	0	0.10	0.743
2	Tmg + group + class	9.7	-3458.9	6937.06	0.22	0.09	0.743
3	Tmg	9.9	-3458.8	6937.26	0.41	0.08	0.743
4	Tmg + class	9.9	-3458.7	6937.3	0.45	0.08	0.743
5	Tmg + group + season	10.4	-3458.3	6937.36	0.51	0.08	0.744
6	Tmg + group + class + season	10.6	-3458.2	6937.57	0.73	0.07	0.743
7	Tmg + season	10.8	-3458.1	6937.76	0.91	0.07	0.743
8	Tmg + class + season	10.9	-3458.0	6937.8	0.95	0.06	0.743
9	Tmg + time + group + season	11.4	-3457.6	6938.12	1.27	0.05	0.744
10	Tmg + time + group + class + season	11.6	-3457.5	6938.33	1.48	0.05	0.744
11	Tmg + time + group	10.5	-3458.8	6938.48	1.63	0.05	0.743
12	Tmg + time + + season	11.8	-3457.4	6938.51	1.66	0.05	0.744
13	Tmg + time + class + season	11.9	-3457.4	6938.54	1.7	0.04	0.743
14	Tmg + time + group + class	10.7	-3458.7	6938.69	1.84	0.04	0.743
15	Tmg + time	10.9	-3458.6	6938.88	2.03	0.04	0.743
16	Tmg + time + class	10.9	-3458.5	6938.92	2.08	0.04	0.743
17	time + group + season	10.7	-3591.2	7203.78	266.93	0.00	0.710
18	time + group + class + season	10.8	-3591.1	7203.88	267.03	0.00	0.710
19	time + season	10.8	-3591.1	7203.93	267.08	0.00	0.710
20	time + class + season	10.9	-3591.1	7203.95	267.11	0.00	0.710
21	group + season	6.8	-3686.4	7386.4	449.56	0.00	0.687
22	group + class + season	6.9	-3686.4	7386.51	449.66	0.00	0.687
23	season	6.9	-3686.4	7386.55	449.7	0.00	0.687
24	class + season	6.9	-3686.4	7386.58	449.73	0.00	0.687
25	time + group	9.3	-3770.3	7559.23	622.39	0.00	0.653
26	time + group + class	9.4	-3770.3	7559.35	622.51	0.00	0.654
27	time	9.5	-3770.2	7559.42	622.57	0.00	0.653
28	time + class	9.5	-3770.2	7559.45	622.61	0.00	0.653
29	group	5.7	-3858.2	7727.77	790.92	0.00	0.618
30	group + class	5.8	-3858.1	7727.89	791.04	0.00	0.618
31	class	5.9	-3858.1	7727.99	791.14	0.00	0.618

Notes: For each model, the model number (no.), number of parameters (K), loglikelihood value (LogLik), Akaike information criteria (AIC_i), difference in AIC between the best fit model and $model_i$ (ΔAIC_i), Akaike weight (w_i), area under receivers operating characteristic curve (ROC) are shown. Parameters: Tmg = black mini-globe temperature; time = time of day; class= age class; group = family group.

S4 Table C. Summary of candidate generalised additive mixed models for the duration of shade use.

no.	model$_i$	K	LogLik	AIC$_i$	ΔAIC$_i$	w_i	R^2
1	Tmg + state + group	7	-879.7	1773.34	0.00	0.37	0.54
2	Tmg + state + group + class	8	-879.4	1774.73	1.39	0.18	0.53
3	Tmg + state + group + season	8	-879.5	1774.94	1.60	0.17	0.54
4	Tmg + state + group + season + class	9	-879.2	1776.48	3.14	0.08	0.53
5	Tmg + time + state + group	9	-879.3	1776.62	3.27	0.07	0.54
6	Tmg + time + state + group + class	10	-879.0	1777.99	4.65	0.04	0.54
7	Tmg + state	6	-883.4	1778.74	5.40	0.02	0.48
8	Tmg + time + state + group + season	10	-879.4	1778.85	5.51	0.02	0.54
9	Tmg + state + season	7	-883.1	1780.25	6.91	0.01	0.47
10	Tmg + time + state + group + season + class	11	-879.2	1780.34	7.00	0.01	0.54
11	Tmg + state + class	7	-883.3	1780.58	7.23	0.01	0.48
12	Tmg + time + state	8	-883.0	1782.07	8.73	0.00	0.48
13	Tmg + state + season + class	8	-883.1	1782.13	8.79	0.00	0.47
14	Tmg + time + state + class	9	-883.0	1783.91	10.57	0.00	0.48
15	Tmg + time + state + season	9	-883.2	1784.31	10.97	0.00	0.47
16	Tmg + time + state + season + class	10	-883.1	1786.17	12.83	0.00	0.47
17	Tmg + group	6	-911.5	1834.99	61.65	0.00	0.37
18	state + time + group + season	8	-909.7	1835.33	61.99	0.00	0.36
19	Tmg + group + class	7	-910.7	1835.48	62.14	0.00	0.37
20	Tmg + group + season	7	-911.6	1837.28	63.94	0.00	0.37
21	time + state + group + season + class	9	-909.7	1837.48	64.14	0.00	0.36
22	Tmg + group + season + class	8	-910.9	1837.74	64.40	0.00	0.37
23	Tmg + time + group	8	-911.5	1839.02	65.68	0.00	0.37
24	state + time + season	7	-912.6	1839.28	65.94	0.00	0.29
25	Tmg + time + group + class	9	-910.7	1839.48	66.14	0.00	0.37
26	Tmg	5	-915.1	1840.25	66.91	0.00	0.30
27	Tmg + time + group + season	9	-911.6	1841.25	67.91	0.00	0.37
28	time + state + season + class	8	-912.7	1841.34	68.00	0.00	0.29
29	Tmg + time + group + season + class	10	-910.8	1841.67	68.33	0.00	0.37
30	Tmg + class	6	-915.0	1841.92	68.58	0.00	0.30
31	Tmg + season	6	-915.3	1842.55	69.21	0.00	0.30
32	Tmg + season + class	7	-915.1	1844.23	70.89	0.00	0.30
33	Tmg + time	7	-915.1	1844.28	70.94	0.00	0.30
34	Tmg + time + class	8	-915.0	1845.95	72.61	0.00	0.30
35	Tmg + time + season	8	-915.3	1846.55	73.21	0.00	0.30
36	Tmg + time + season + class	9	-915.1	1848.23	74.89	0.00	0.30
37	state + group + season	6	-924.8	1861.59	88.25	0.00	0.27
38	state + group + season + class	7	-924.8	1863.67	90.33	0.00	0.27
39	group + time + season	7	-925.0	1863.98	90.64	0.00	0.26
40	state + season	5	-927.4	1864.86	91.52	0.00	0.19
41	time + group + season + class	8	-924.9	1865.83	92.48	0.00	0.26
42	state + season + class	6	-927.4	1866.89	93.55	0.00	0.19

Summary of candidate models for the duration of shade use continued...

no.	$model_i$	K	LogLik	AIC_i	ΔAIC_i	w_i	R^2
43	time + season	6	-928.1	1868.10	94.76	0.00	0.18
44	time + season + class	7	-928.0	1870.05	96.71	0.00	0.18
45	state + time + group	7	-929.6	1873.19	99.85	0.00	0.19
46	time + state + group + class	8	-929.5	1875.02	101.68	0.00	0.19
47	time + state	6	-932.2	1876.39	103.05	0.00	0.12
48	time + state + class	7	-932.2	1878.30	104.96	0.00	0.12
49	state + group	5	-934.5	1878.98	105.64	0.00	0.10
50	state + group + class	6	-934.4	1880.89	107.55	0.00	0.10
51	state	4	-936.8	1881.51	108.17	0.00	0.03
52	time + group	6	-935.4	1882.85	109.51	0.00	0.13
53	group + season	5	-936.7	1883.41	110.07	0.00	0.20
54	state + class	5	-936.8	1883.69	110.35	0.00	0.03
55	time + group + class	7	-935.4	1884.81	111.46	0.00	0.13
56	group + season + class	6	-936.6	1885.24	111.90	0.00	0.20
57	time	5	-938.1	1886.15	112.81	0.00	0.06
58	season	5	-939.4	1886.77	113.42	0.00	0.12
59	time + class	6	-938.2	1888.41	115.07	0.00	0.07
60	season + class	5	-939.6	1889.13	115.79	0.00	0.11
61	group	4	-942.2	1892.43	119.09	0.00	0.07
62	group + class	5	-942.2	1894.32	120.98	0.00	0.07
63	class	4	-944.7	1897.41	124.07	0.00	0.00

Notes: For each model the number of parameters (K), loglikelihood value (LogLik), difference in AIC between the best fit model and $model_i$ (ΔAIC_i), Akaike weight (w_i), adjusted coefficient of determination (R^2) are shown. Parameters: Tmg = black mini-globe temperature; time = time of day; class= age class; group = family group; state = whether elephant was wet or dry.

S4 Table D. Summary of candidate generalised additive mixed models for the probability of drinking.

no.	$model_i$	K	LogLik	AIC_i	ΔAIC_i	w_i	ROC
1	Tmg + time	4.3	-492.3	993.23	0	0.21	0.644
2	Tmg + time + class	5.3	-491.4	993.54	0.31	0.18	0.650
3	Tmg + time + season	5.3	-491.8	994.16	0.93	0.13	0.645
4	Tmg + time + class + season	6.3	-490.8	994.18	0.95	0.13	0.650
5	time	2.0	-495.2	994.46	1.23	0.12	0.629
6	time + class	3.0	-494.4	994.75	1.51	0.10	0.634
7	time + season	3.0	-494.9	995.81	2.58	0.06	0.631
8	time + class + season	4.0	-493.9	995.89	2.65	0.06	0.637
9	Tmg	3.1	-500.8	1007.79	14.56	0.00	0.570
10	Tmg + class	4.1	-500.0	1008.17	14.94	0.00	0.585
11	Tmg + season	4.3	-500.4	1009.22	15.99	0.00	0.580
12	Tmg + class + season	5.2	-499.7	1009.78	16.55	0.00	0.591
13	class	2.0	-503.9	1011.88	18.65	0.00	0.554
14	season	2.0	-504.4	1012.88	19.65	0.00	0.551
15	class + season	3.0	-503.5	1013.07	19.84	0.00	0.556

Notes: For each model, the model number (no.), number of parameters (K), loglikelihood value (LogLik), Akaike information criteria (AIC_i), difference in AIC between the best fit model and $model_i$ (ΔAIC_i), Akaike weight (w_i), area under receivers operating characteristic curve (ROC) are shown. Parameters: Tmg = black mini-globe temperature; time = time of day; class= age class; group = family group.

S4 Table E. Summary of candidate generalised additive mixed models for the probability of walking.

no.	$model_i$	K	LogLik	AIC_i	ΔAIC_i	w_i	ROC
1	Tmg + time + group	9.5	-1528.09	3075.26	0.00	0.52	0.622
2	Tmg + time + group + season	10.4	-1527.98	3076.78	1.52	0.24	0.624
3	Tmg + time	12.0	-1526.86	3077.66	2.40	0.16	0.626
4	Tmg + time + season	13.0	-1526.68	3079.35	4.09	0.07	0.627
5	Tmg + group	4.9	-1536.98	3083.78	8.52	0.01	0.599
6	Tmg + group + season	5.8	-1537.01	3085.69	10.43	0.00	0.599
7	Tmg	7.3	-1535.73	3086.07	10.81	0.00	0.605
8	Tmg + season	8.3	-1535.72	3088.02	12.76	0.00	0.605
9	time + group + season	8.6	-1537.09	3091.34	16.08	0.00	0.600
10	time + + season	10.4	-1536.24	3093.33	18.07	0.00	0.606
11	time + group	6.8	-1541.69	3096.99	21.73	0.00	0.587
12	time	9.4	-1540.5	3099.87	24.61	0.00	0.595
13	group + season	4.0	-1548.12	3104.24	28.98	0.00	0.574
14	season	5.8	-1547.26	3106.17	30.91	0.00	0.577
15	group	2.2	-1552.97	3110.26	35.00	0.00	0.564

Notes: For each model, the model number (no.), number of parameters (K), loglikelihood value (LogLik), Akaike information criteria (AIC_i), difference in AIC between the best fit model and $model_i$ (ΔAIC_i), Akaike weight (w_i), area under receivers operating characteristic curve (ROC) are shown. Parameters: Tmg = black mini-globe temperature; time = time of day; class= age class; group = family group.

S4 Table F. Summary of candidate generalised additive mixed models for the probability of resting.

no.	$model_i$	K	LogLik	AIC_i	ΔAIC_i	w_i	ROC
1	Tmg	7.5	-557.83	1130.65	0	0.10	0.788
2	Tmg + class	7.6	-557.85	1130.86	0.21	0.09	0.789
3	Tmg + group	7.6	-557.81	1130.89	0.24	0.09	0.788
4	Tmg + time	9.8	-555.69	1130.98	0.33	0.09	0.793
5	Tmg + group + class	7.7	-557.81	1131.08	0.43	0.08	0.789
6	Tmg + time + class	9.9	-555.71	1131.18	0.53	0.08	0.793
7	Tmg + time + group	9.9	-555.67	1131.21	0.56	0.08	0.793
8	Tmg + time + group + class	10.	-555.66	1131.40	0.75	0.07	0.794
9	Tmg + season	8.6	-557.33	1131.85	1.2	0.06	0.789
10	Tmg + class + season	8.7	-557.34	1132.05	1.4	0.05	0.790
11	Tmg + group + season	8.7	-557.32	1132.10	1.45	0.05	0.789
12	Tmg + group + class + season	8.8	-557.31	1132.28	1.63	0.04	0.789
13	Tmg + time + season	10.8	-555.56	1132.63	1.98	0.04	0.793
14	Tmg + time + class + season	10.8	-555.59	1132.84	2.19	0.03	0.792
15	Tmg + time + group + season	10.9	-555.54	1132.87	2.21	0.03	0.793
16	Tmg + time + group + class + season	11	-555.54	1133.05	2.4	0.03	0.793
17	time + season	9.5	-566.68	1152.31	21.66	0.00	0.781
18	time + group + season	9.6	-566.67	1152.51	21.86	0.00	0.781
19	time + class + season	9.6	-566.68	1152.53	21.88	0.00	0.781
20	time + group + class + season	9.7	-566.64	1152.71	22.06	0.00	0.781
21	season	6.5	-576.43	1165.81	35.16	0.00	0.752
22	group + season	6.6	-576.42	1166.01	35.36	0.00	0.752
23	class + season	6.6	-576.43	1166.03	35.38	0.00	0.752
24	group + class + season	6.7	-576.4	1166.21	35.56	0.00	0.752
25	time	8.4	-577.86	1172.58	41.93	0.00	0.757
26	time + class	8.5	-577.86	1172.78	42.13	0.00	0.757
27	time + group	8.6	-577.84	1172.79	42.14	0.00	0.757
28	time + group + class	8.7	-577.83	1172.96	42.31	0.00	0.755
29	class	5.6	-587.46	1186.03	55.38	0.00	0.716
30	group	5.6	-587.45	1186.05	55.4	0.00	0.716
31	group + class	5.7	-587.43	1186.23	55.57	0.00	0.716

Notes: For each model, the model number (no.), number of parameters (K), loglikelihood value (LogLik), Akaike information criteria (AIC_i), difference in AIC between the best fit model and $model_i$ (ΔAIC_i), Akaike weight (w_i), area under receivers operating characteristic curve (ROC) are shown. Parameters: Tmg = black mini-globe temperature; time = time of day; class= age class; group = family group.

S4 Table G. Summary of candidate generalised additive mixed models for the probability of feeding.

no.	$model_i$	K	LogLik	AIC_i	ΔAIC_i	w_i	ROC
1	Tmg + time + season	12.2	-2794.1	5612.69	0	0.47	0.636
2	Tmg + time + class + season	12.4	-2794.0	5612.91	0.22	0.42	0.636
3	Tmg + season	9.7	-2799.0	5617.43	4.74	0.04	0.634
4	Tmg + class + season	9.9	-2798.9	5617.66	4.97	0.04	0.634
5	Tmg + time	10.9	-2799.0	5619.79	7.1	0.01	0.634
6	Tmg + time + class	11.2	-2798.8	5620.01	7.31	0.01	0.634
7	Tmg	8.9	-2804.5	5626.93	14.24	0.00	0.629
8	Tmg + class	9.2	-2804.4	5627.16	14.47	0.00	0.629
9	time + season	9.9	-2814.3	5648.29	35.6	0.00	0.620
10	time + class + season	10.1	-2814.2	5648.51	35.81	0.00	0.620
11	season	6.5	-2829.8	5672.45	59.76	0.00	0.610
12	class + season	6.7	-2829.7	5672.67	59.98	0.00	0.610
13	time	8.8	-2862.0	5741.70	129.01	0.00	0.581
14	time + class	9.0	-2861.9	5741.93	129.24	0.00	0.581
15	class	5.6	-2876.7	5764.46	151.76	0.00	0.559

Notes: For each model, the model number (no.), number of parameters (K), loglikelihood value (LogLik), Akaike information criteria (AIC_i), difference in AIC between the best fit model and $model_i$ (ΔAIC_i), Akaike weight (w_i), area under receivers operating characteristic curve (ROC) are shown. Parameters: Tmg = black mini-globe temperature; time = time of day; class= age class; group = family group.

S4 Table H. Summary of candidate generalised additive mixed models for variation in skin temperature for seven elephants.

	Candidate model	K	LogLik	AIC	Δ AIC_i	w_i	R^2
1	mini-globe + state + age class	10	-18526	37072	0	0.86	0.36
2	mini-globe + state	8	-18529	37075	3.59	0.14	0.32
3	mini-globe + age class	8	-19019	38053	981.91	0	0.26
4	mini-globe	6	-19022	38057	984.97	0	0.21
5	age class + state	8	-20139	40293	3221.88	0	0.07
6	State	6	-20142	40297	3224.97	0	0.03
7	age class	6	-20263	40539	3467.41	0	0.04

Note: K=number of parameters in model; LogLik= log likelihood; AIC= Akaike's information criterion; Δ AIC_i= Difference in AIC between the model and best fitting model; w_i=Akaike weight; R^2=adjusted coefficient of determination. Parameters: mini-globe = black mini-globe temperature; state = whether elephant is wet or dry.

S4 Table I. Summary of candidate generalised additive mixed models for variation in core temperature for five elephants.

	Candidate model	K	LogLik	AIC	Δ AIC_i	w_i	R^2
1	mini-globe + time + age class	8	-60.98	137.97	0.00	0.49	0.31
2	mini-globe + time	7	-62.25	138.49	0.52	0.38	0.18
3	time + age class	6	-64.89	141.77	3.80	0.07	0.30
4	time	5	-66.23	142.46	4.49	0.05	0.17
5	mini-globe + time + age class + state	10	-65.21	150.42	12.46	0.00	0.31
6	mini-globe + time + state	9	-66.49	150.98	13.01	0.00	0.18
7	time + age class + state	8	-69.72	155.45	17.48	0.00	0.30
8	time + state	7	-71.08	156.17	18.20	0.00	0.17
9	mini-globe + age class	6	-112.54	237.08	99.11	0.00	0.19
10	mini-globe + age class + state	6	-112.54	237.08	99.11	0.00	0.19
11	mini-globe	5	-113.77	237.55	99.58	0.00	0.07
12	mini-globe + state	7	-117.47	248.93	110.97	0.00	0.08
13	age class	4	-133.24	274.47	136.51	0.00	0.13
14	age class + state	6	-136.98	285.97	148.00	0.00	0.12
15	state	5	-138.28	286.56	148.59	0.00	-0.01

Note: K=number of parameters in model; LogLik= log likelihood; AIC= Akaike's information criterion; Δ AIC_i= Difference in AIC between the model and best fitting model; w_i=Akaike weight; R^2=adjusted coefficient of determination. Parameters: mini-globe = black mini-globe temperature; time = time of day; state = whether elephant is wet or dry.
